# Supplementary material for: APOE deficiency impacts neural differentiation and cholesterol biosynthesis in human iPSC-derived cerebral organoids
Source: Stem Cell Res Ther. 2023 Aug 21;14:214. doi: 10.1186/s13287-023-03444-y (PMC10441762; doi:10.1186/s13287-023-03444-y)
Supplement: Supplementary file 1 — Additional file 1: Fig. S1. Marker gene expression in iPSC-derived cerebral organoids. a The schematic shows the workflow of scRNA-seq analysis of cerebral organoids and related validation. b–i t-SNE plots indicating the expression of key marker genes for different cell types. oRG, outer radial glia; IPC, intermediate progenitor cell; ExN, excitatory neuron; InN, inhibitory neuron. Fig. S2. APOE expression and distribution in the iPSC-derived cerebral organoids. a Violin plot of APOE expression in different cell clusters of both control and APOE-/- cerebral organoids. b Representative images of immunostaining for apoE and an astrocytic marker S100β in cerebral organoids. Scale bar: 200 µm. Fig. S3. Differentiation pattern changes in the APOE-deficient cerebral organoids. a t-SNE plots for cerebral layer markers (BCL11B, SATB2) and astrocytic markers (S100B, GFAP) in the control and APOE-/- cerebral organoids. b Pseudotime trajectory analysis of the cerebral organoids. Cells (dots) are colored according to pseudotime, from origin in dark purple to terminal state in light yellow. c Cortical and hippocampal identity evaluation in neuronal clusters via analysis of signature genes expression. Fig. S4. DEGs and pathway analysis for each excitatory neuron cluster. Volcano plots for DEGs and gene ontology analyses in excitatory neuron cluster 0 (a, b), cluster 1 (c, d), cluster 2 (e, f), and cluster 5 (g, h). Fig. S5. Changes in the expression of cholesterol biosynthesis-related genes in different cell types within the iPSC-derived cerebral organoids. a Schematic diagram for cholesterol biosynthesis in Bloch pathway. b–d Expression of specific cholesterol biosynthetic genes in excitatory neurons cluster 3 (b), radial glia (c) and astrocytes (d) from the control and APOE-/- cerebral organoids are visualized in a heatmap. Fig. S6. Confirmation of key findings using another set of APOE-deficient iPSC-derived cerebral organoids. Another APOE-deficient iPSC line (MC0192-4C11) wa [file 13287_2023_3444_MOESM1_ESM.docx]

**Supplementary information**


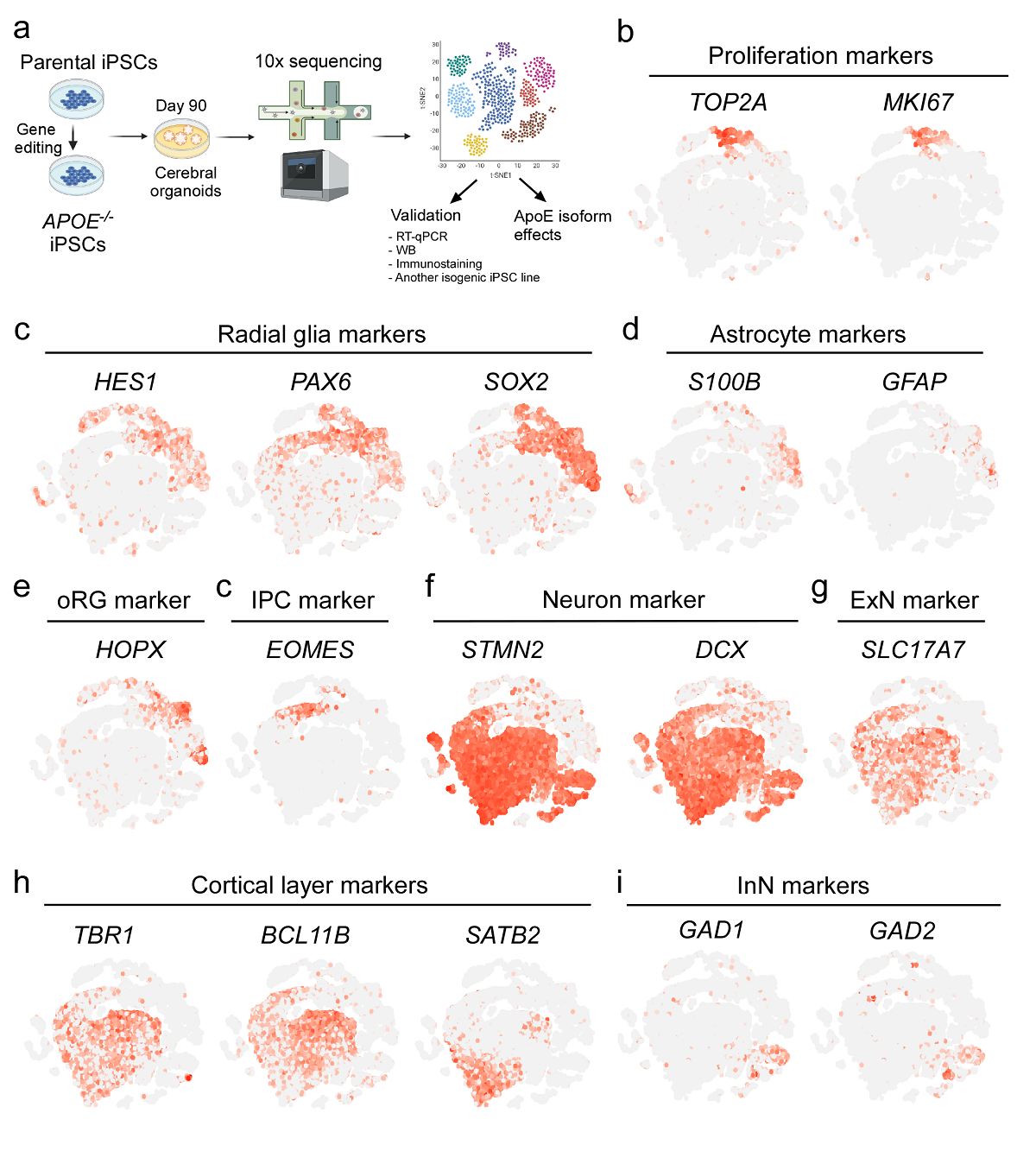


**Supplementary Figure 1.** **Marker gene expression in iPSC-derived cerebral organoids.**

**a** The schematic shows the workflow of scRNA-seq analysis of cerebral organoids and related validation. **b-i** t-SNE plots indicating the expression of key marker genes for different cell types. oRG, outer radial glia; IPC, intermediate progenitor cell; ExN, excitatory neuron; InN, inhibitory neuron.

**
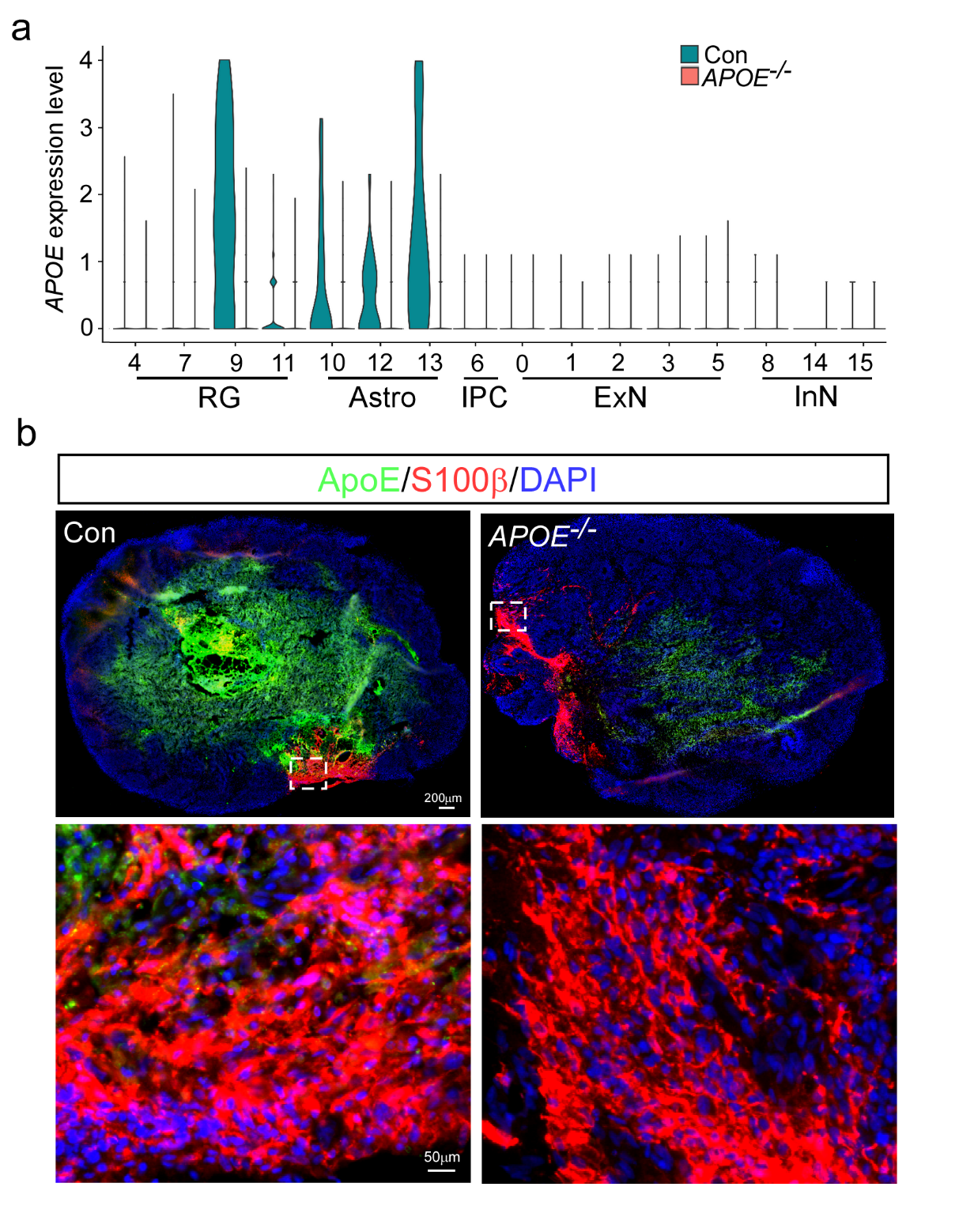
**

**Supplementary Figure 2. *APOE* expression and distribution in the iPSC-derived cerebral organoids.**

**a** Violin plot of *APOE* expression in different cell clusters of both control and *APOE^-/-^* cerebral organoids. **b** Representative images of immunostaining for apoE and an astrocytic marker S100β in cerebral organoids. Scale bar: 200 μm.

**
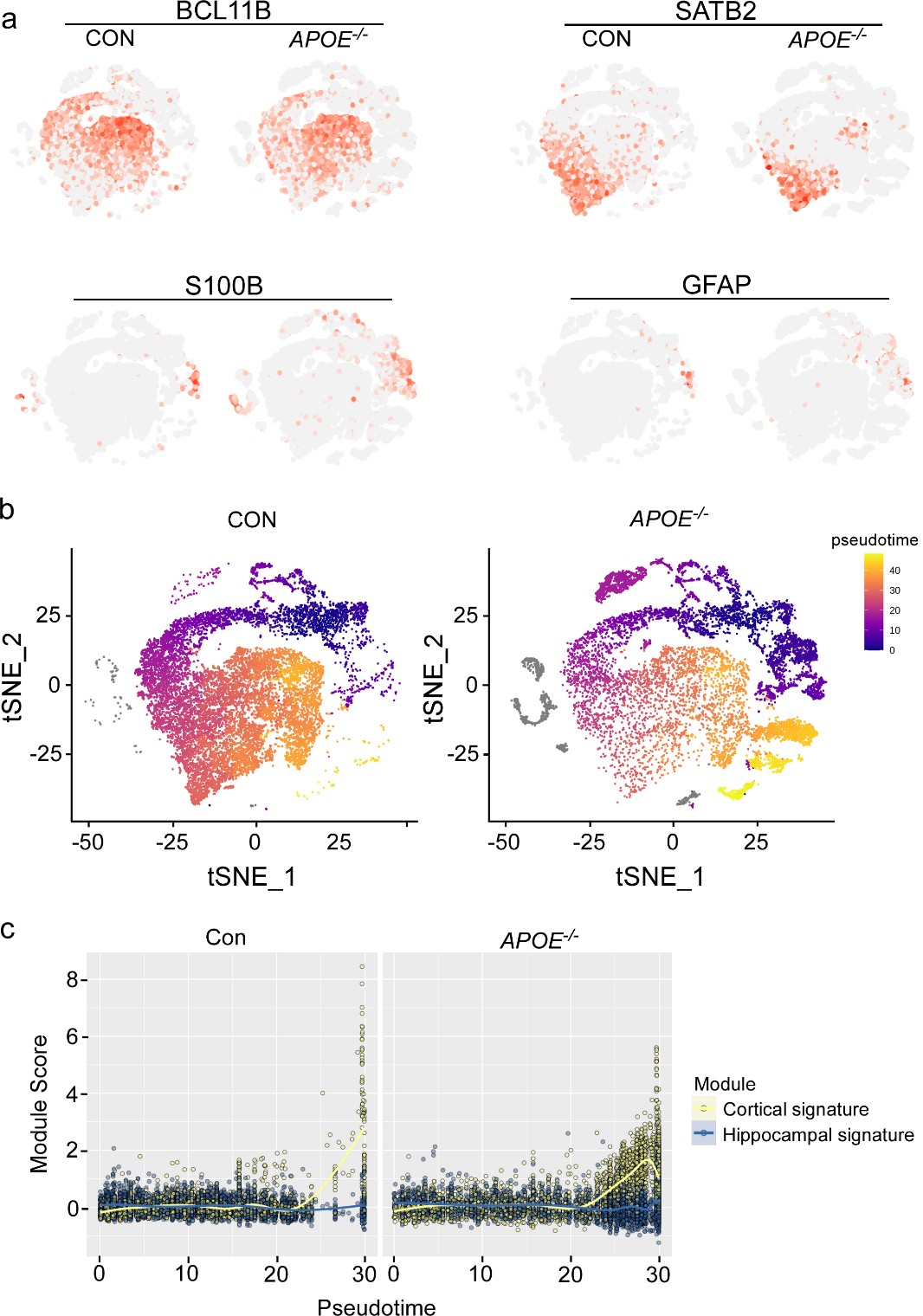
**

**Supplementary Figure 3. Differentiation pattern changes in the *APOE* deficient cerebral organoids.**

**a** t-SNE plots for cerebral layer markers (*BCL11B*, *SATB2*) and astrocytic markers (*S100B*, *GFAP*) in the control and *APOE^-/-^* cerebral organoids. **b** Pseudotime trajectory analysis of the cerebral organoids. Cells (dots) are colored according to pseudotime, from origin in dark purple to terminal state in light yellow. **c** Cortical and hippocampal identity evaluation in neuronal clusters via analysis of signature genes expression.

**
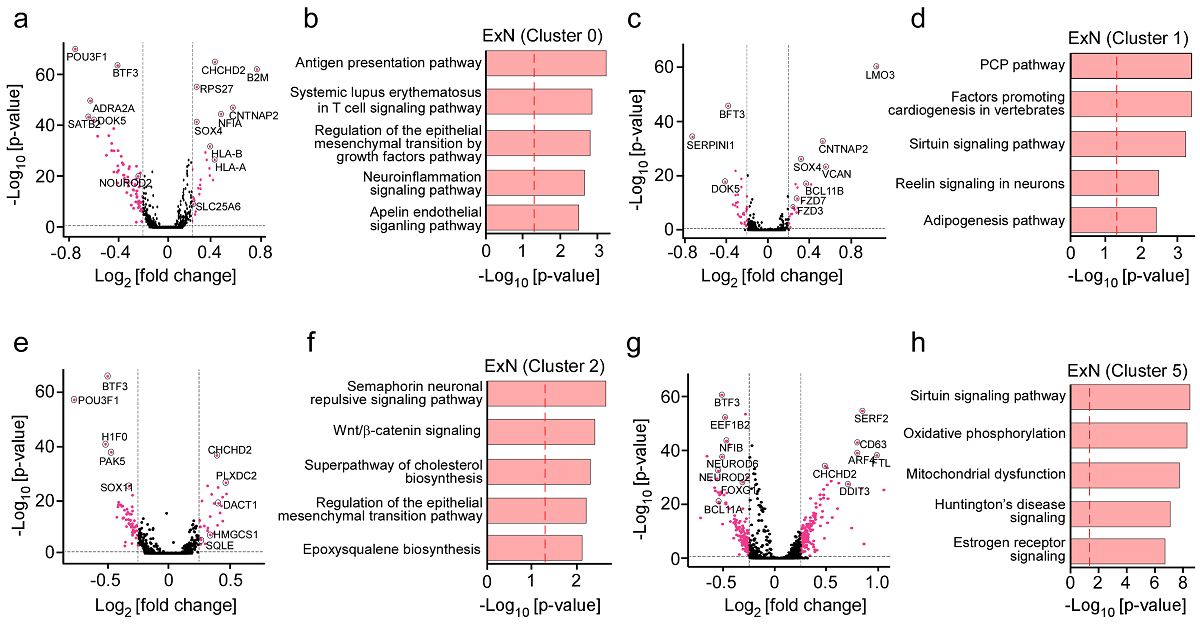
**

**Supplementary figure 4.** **DEGs and pathway analysis for each excitatory neuron cluster.**

Volcano plots for DEGs and gene ontology analyses in excitatory neuron cluster 0 (a, b), cluster 1 (c, d), cluster 2 (e, f), and cluster 5 (g, h).

**
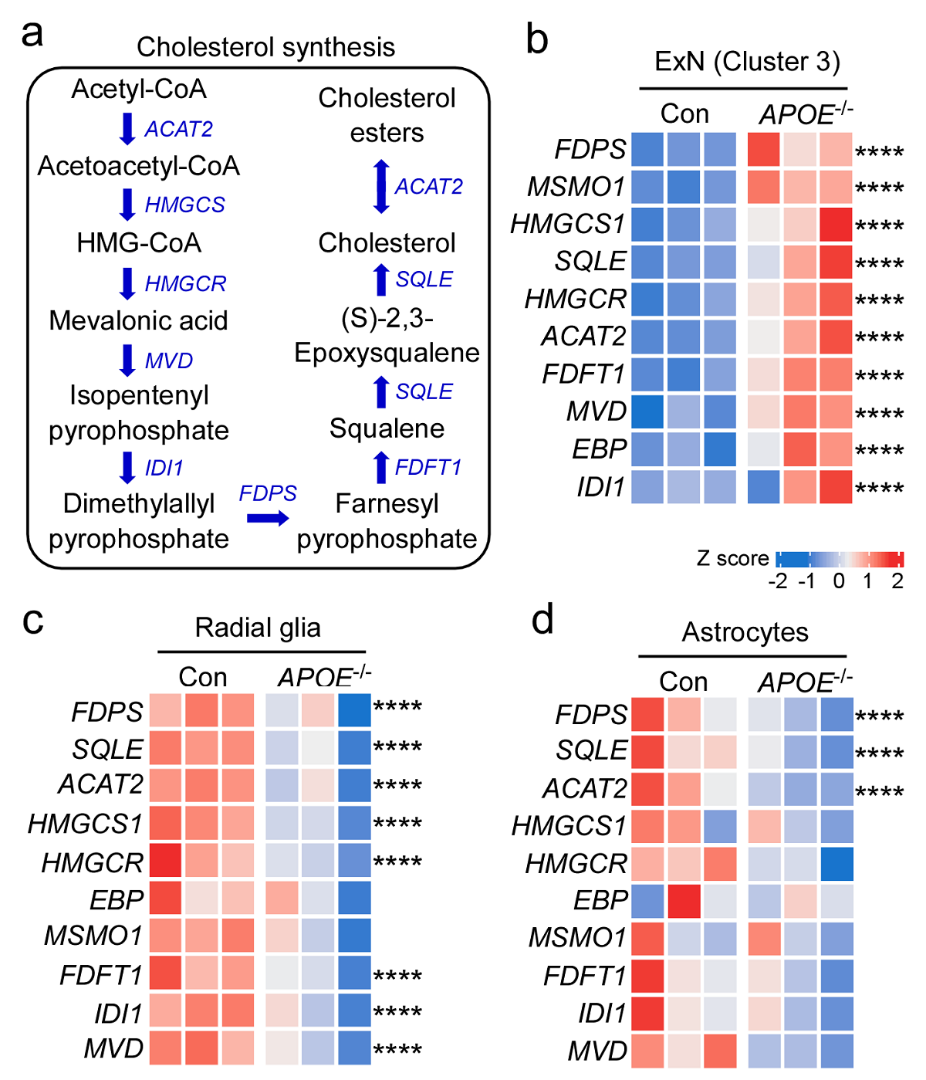
**

**Supplementary Figure 5. Changes in the expression of cholesterol biosynthesis-related genes in different cell types within the iPSC-derived cerebral organoids.**

**a** Schematic diagram for cholesterol biosynthesis in Bloch pathway. **b-d** Expression of specific cholesterol biosynthetic genes in excitatory neurons cluster 3 (b), radial glia (c) and astrocytes (d) from the control and *APOE^-/-^* cerebral organoids are visualized in a heatmap.

**
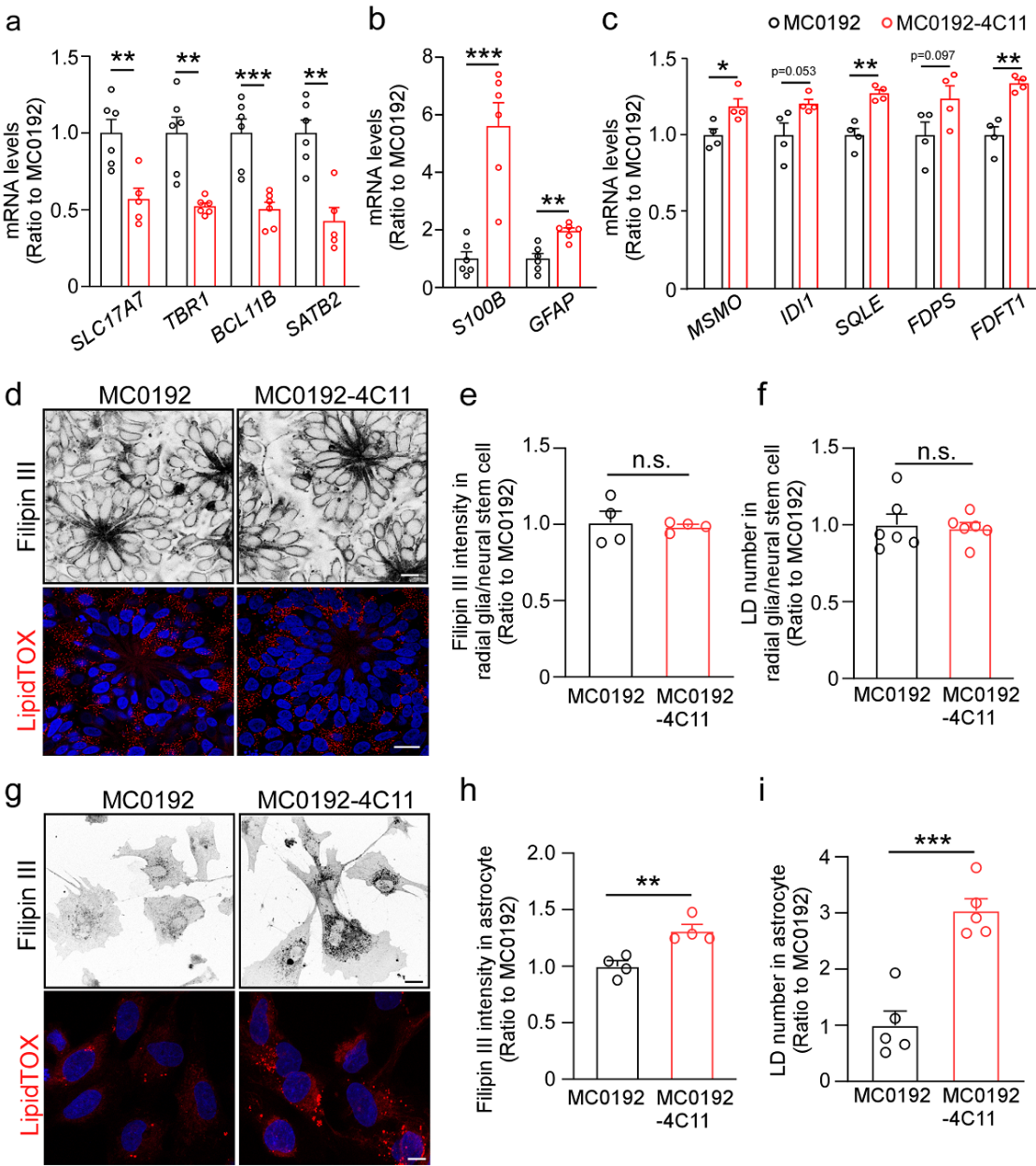
**

**Supplementary Figure 6.** **Confirmation of key findings using another set of *APOE* deficient iPSC-derived cerebral organoids.**

Another *APOE* deficient iPSC line (MC0192-4C11) was generated from a control iPSC line (MC0192). The parent control and isogenic *APOE* deficient iPSCs were differentiated into cerebral organoids and subjected to analysis at Day 90. **a, b** The mRNA levels of cerebral layer markers (a; *SLC17A7*, *TBR1*, *BCL11B* and *SATB2*) and astrocytic markers (b; *S100B* and *GFAP*) were quantified by RT-qPCR. Three cerebral organoids were pooled and analyzed as one sample (n=6 samples/genotype). **c** The mRNA levels of selective cholesterol biosynthesis genes in neurons isolated from the cerebral organoids were quantified by RT-qPCR (n=4 wells/genotype). **d-f** The radial glia/neural stem cells differentiated from the iPSCs were plated on coverslips and stained with Filipin III and LipidTOX (d). Filipin III intensities (e) and lipid droplet number (f) were quantified in 3 fields of each coverslip and averaged (n=4-6 coverslips/genotype). Scale bars, 10 μm. **g-i** The isolated astrocytes from the iPSCs were plated on coverslips and stained with Filipin III and LipidTOX (g). Filipin III intensities (h) and lipid droplet number (i) were quantified in 3 fields of each coverslip and averaged (n=4-5 coverslips/genotype). Scale bars: 10 μm. Experiments were repeated in two independently differentiated batches. All data are expressed as mean ± SEM. Student’s t tests were performed to determine statistical significance. *p<0.05, **p<0.01, *** p<0.001, ****p<0.0001.


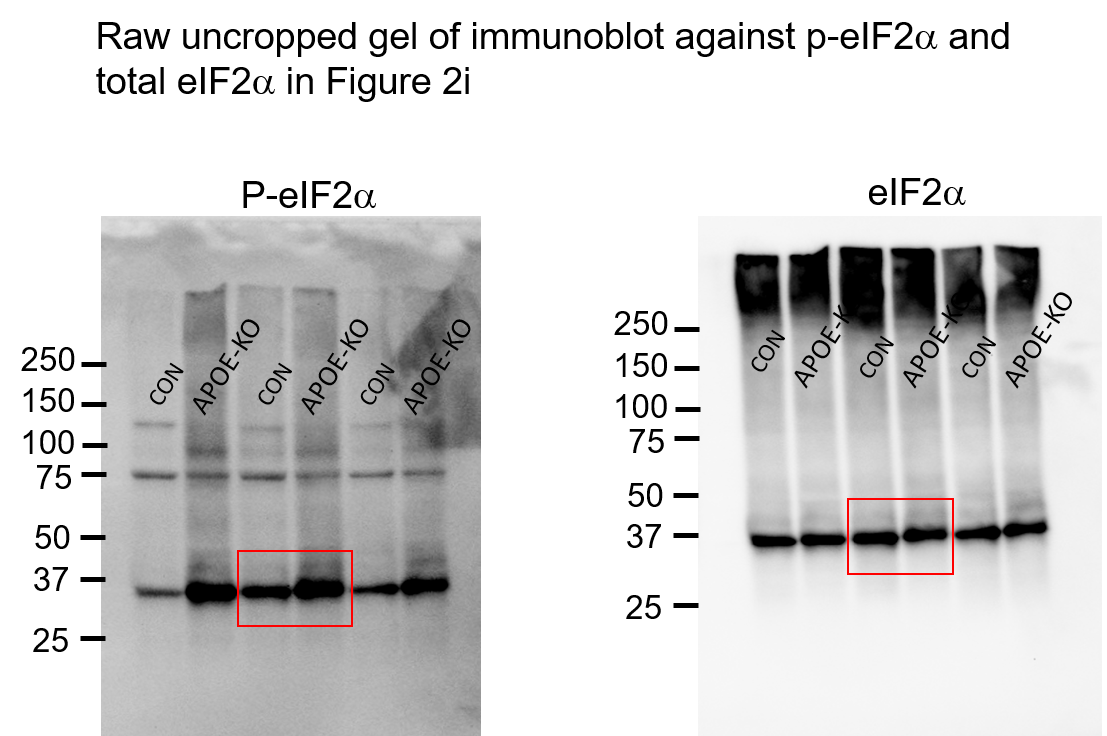


**Supplementary Figure 7. Raw uncropped gel of immunoblot against p-eIF2α and total eIF2α in Figure 2i.**


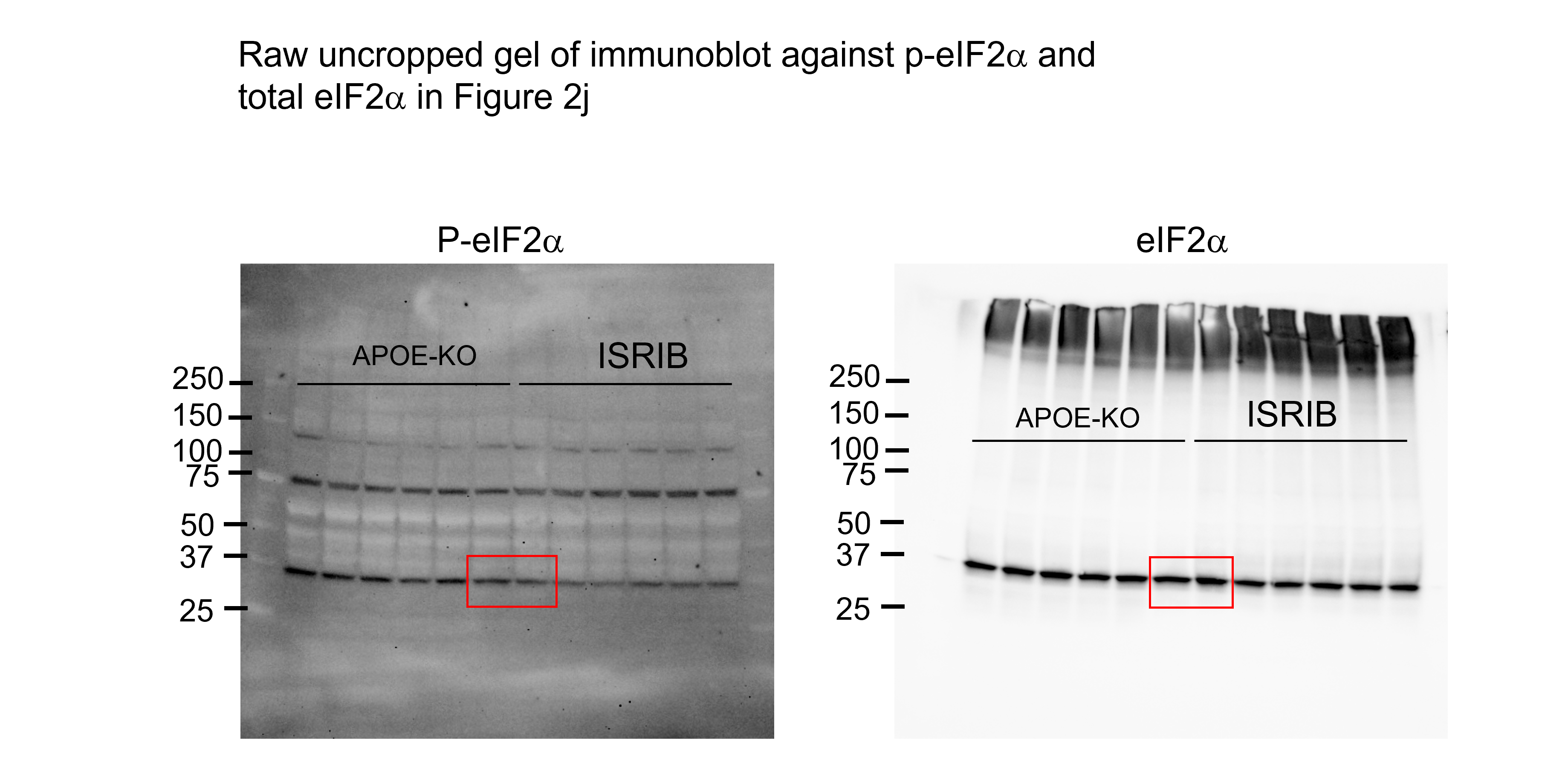


**Supplementary Figure 8. Raw uncropped gel of immunoblot against p-eIF2α and total eIF2α in Figure 2j.**


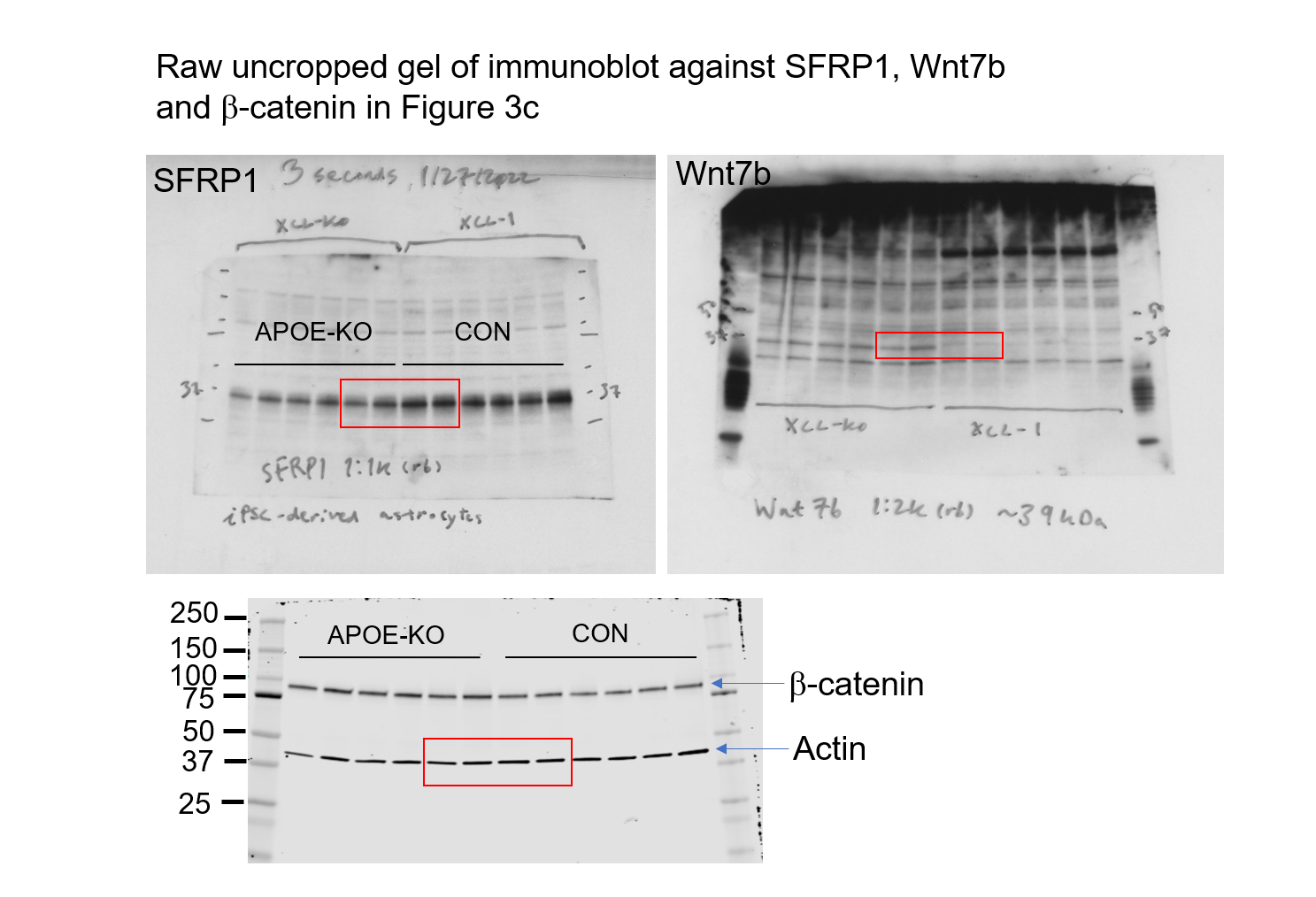


**Supplementary Figure 9. Raw uncropped gel of immunoblot against SFRP1, Wnt7b and β-catenin in Figure 3c.**


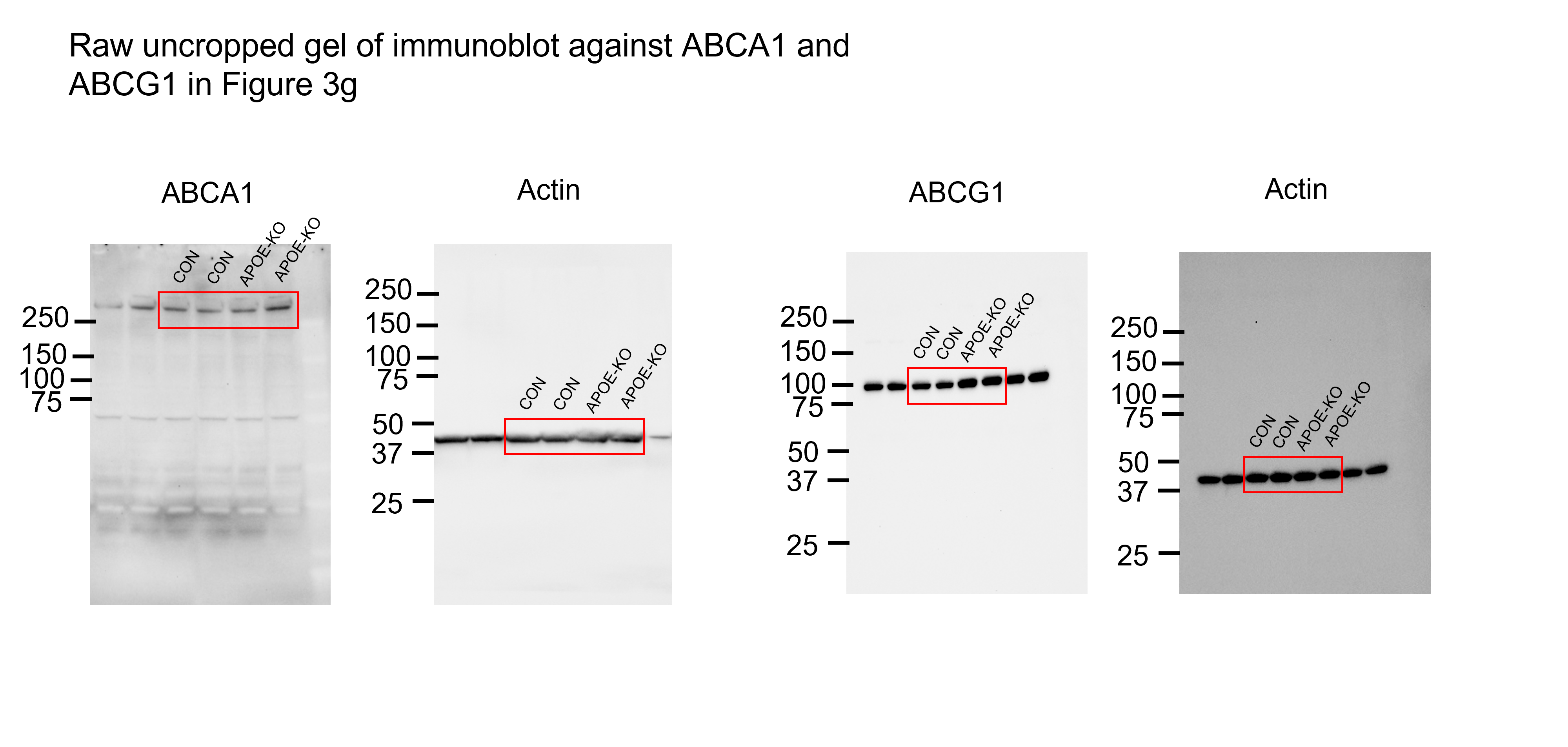


**Supplementary Figure 10. Raw uncropped gel of immunoblot against ABCA1 and ABCG1 in Figure 3g.**


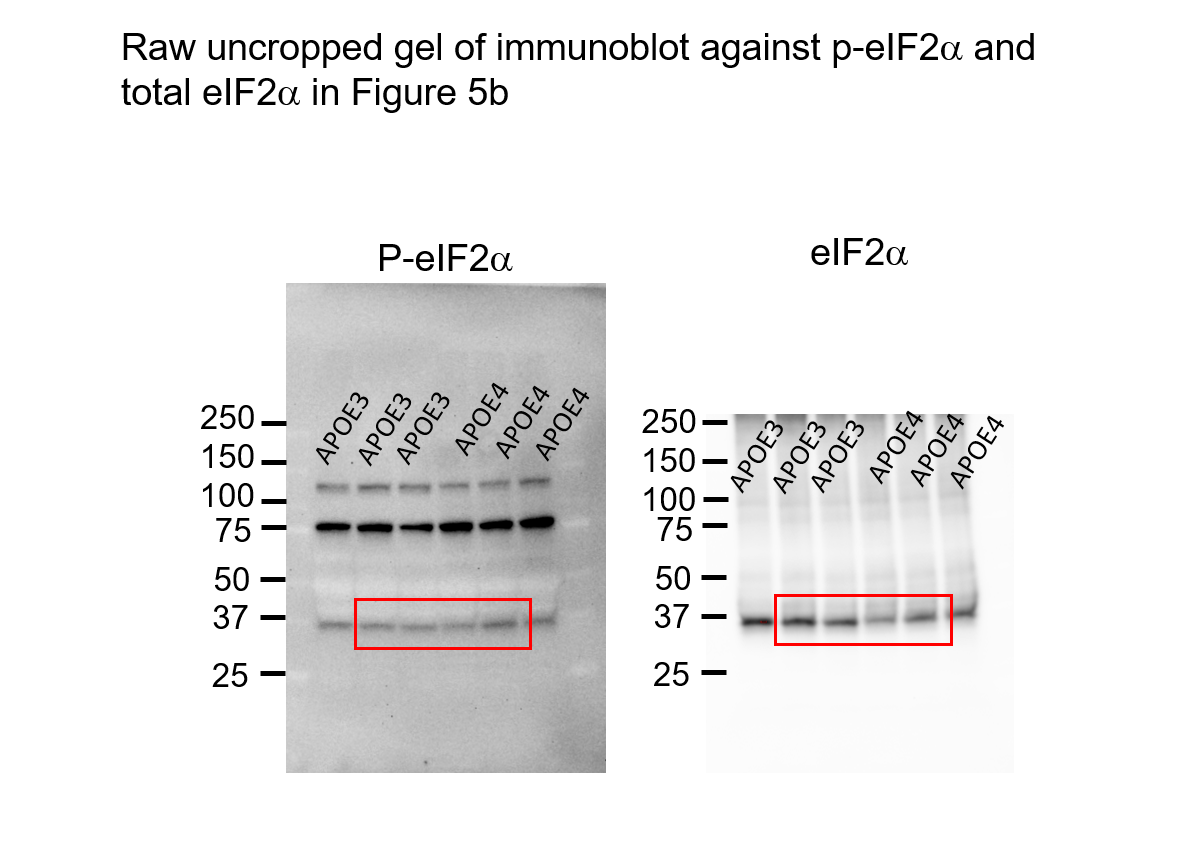


**Supplementary Figure 11. Raw uncropped gel of immunoblot against p-eIF2α and total eIF2α in Figure 5b.**

**Supplementary Table 1. Primer information for RT-qPCR**

| Actin | Forward | 5’-CTGGCACCACACCTTCTACAATG-3’ |
| --- | --- | --- |
|  | Reverse | 5’-AATGTCACGCACGATTTCCCGC-3’ |
| BCL11B | Forward | 5’-GTTGTGCAAATGTAGCTGGAA-3’ |
|  | Reverse | 5’-GAAGATGACCACCTGCTCTC-3’ |
| SATB2 | Forward | 5’-CCTTACGCAGAATCTCAGACAA-3’ |
|  | Reverse | 5’-CCAGATATCTACCAGCAAGTCAG-3’ |
| S100B | Forward | 5’-TCTAACTCAGGACCGAGAATCA-3’ |
|  | Reverse | 5’-GGAGCAAGGAAGATACAACTAACT-3’ |
| GFAP | Forward | 5’-CTGTTGCCAGAGATGGAGGTT-3’ |
|  | Reverse | 5’-TCATCGCTCAGGAGGTCCTT-3’ |
| SLC17A7 | Forward | 5’-CCATGACTAAGCACAAGACTC-3’ |
|  | Reverse | 5’-AGATGACACCTCCATAGTGC-3’ |
| TBR1 | Forward | 5’-CGTGTCATAATTATCCCGAAATCC-3’ |
|  | Reverse | 5’-CAGACGTTCACTTTCCCTGAG-3’ |
| SOX2 | Forward | 5’-CCCAGCAGACTTCACATGT-3’ |
|  | Reverse | 5’-CCTCCCATTTCCCTCGTTTT-3’ |
| HMGCR | Forward | 5’-GTTTACTGGTAACAATAAGATCTGTGGTTGG-3’ |
|  | Reverse | 5’-ACAGGATGGCTATGCATCGTGTTATTGTC-3’ |
| IDI1 | Forward | 5’-TGGATAAAACCCCTGTGGTG-3’ |
|  | Reverse | 5’-CAACATCCGGCATAACTGTG-3’ |
| SQLE | Forward | 5’-ACCCGAGTCCAGTTCTCATCTA-3’ |
|  | Reverse | 5’-CCTTGGCATTTCTCCTCTAATG-3’ |
| FDPS | Forward | 5’-CTGTCCCGCTGGTTGAGATC-3’ |
|  | Reverse | 5’-GTTCATTCTGAGGGAGGAGCAA-3’ |
| FDFT1 | Forward | 5’-AAGATTCGGAAAGGGCAAGC-3’ |
|  | Reverse | 5’-GTCCGGATGGTGGAGATGAT-3’ |
| HMGCS | Forward | 5’-TCGTGGCTCACTCCCTTTCC-3’ |
|  | Reverse | 5’-GGCCAGCAAGCTTCTGCATT-3’ |
| MSMO | Forward | 5’-ATCATGAGTTTCAGGCTCCATT-3’ |
|  | Reverse | 5’-AAGCACGATTCCAATGAAAAAT-3’ |
